# Cancer as a Channelopathy—Appreciation of Complimentary Pathways Provides a Different Perspective for Developing Treatments

**DOI:** 10.3390/cancers14194627

**Published:** 2022-09-23

**Authors:** Harry J. Gould, Dennis Paul

**Affiliations:** 1 Department of Neurology, Louisiana State University Health Sciences Center, New Orleans, LA 70112, USA; 2 Department of Pharmacology and Experimental Therapeutics, Louisiana State University Health Sciences Center, New Orleans, LA 70112, USA

**Keywords:** cancer, channelopathy, targeted osmotic lysis, ion channels, Na^+^, K^+^-ATPase, pulsed electric fields

## Abstract

**Simple Summary:**

While improvements in technology have improved our ability to treat many forms of cancer when diagnosed at an early stage of the disease, the ability to improve survival and quality of life for patients with late stage disease has been limited, largely due to the ability of cancer cells to evade destruction when treatments block preferred paths for survival. Here, we review the role that ions and ion channels play in normal cell function, the development of disease and their role in the life and death of a cell. It is hoped that viewing cancer from the perspective of altered ion channel expression and ion balance may provide a novel approach for developing more effective treatments for this devastating disease.

**Abstract:**

Life depends upon the ability of cells to evaluate and adapt to a constantly changing environment and to maintain internal stability to allow essential biochemical reactions to occur. Ions and ion channels play a crucial role in this process and are essential for survival. Alterations in the expression of the transmembrane proteins responsible for maintaining ion balance that occur as a result of mutations in the genetic code or in response to iatrogenically induced changes in the extracellular environment is a characteristic feature of oncogenesis and identifies cancer as one of a constellation of diseases known as channelopathies. The classification of cancer as a channelopathy provides a different perspective for viewing the disease. Potentially, it may expand opportunities for developing novel ways to affect or reverse the deleterious changes that underlie establishing and sustaining disease and developing tolerance to therapeutic attempts at treatment. The role of ions and ion channels and their interactions in the cell’s ability to maintain ionic balance, homeostasis, and survival are reviewed and possible approaches that mitigate gain or loss of ion channel function to contribute to new or enhance existing cancer therapies are discussed.

## 1. Introduction

For decades, the oncology community has regarded cancer from several viewpoints. Originally thought of only as a disease of uncontrolled growth, it is additionally viewed as a failure of cell death, genetic mutation, or a failure of the immune system. Moreover, metastasis can be thought of as an increase of motility and invasiveness, and recurrence can be viewed as cancer cells exiting an extended G0 phase of the cell cycle or hibernation and a return to the features of stem cells. The viewpoint from which the disease is viewed determines the approaches to treatment that are likely to be conceived.

Louis Ptáček and his colleagues [1,2,3], upon discovering that a mutation in the gene that codes for the expression of the sodium channel in muscle responsible for contraction was the cause of hyperkalemic periodic paralysis, introduced the term, channelopathy, to highlight the importance of altered ion channel functioning in the phenotypic presentation of the disease [4]. Since then, it has been recognized that many other diseases [5,6,7,8,9,10,11], similarly share altered ion channel expression and the resulting determinants of abnormal gain or loss of function associated with mutations in the genetic code or in response to changes in the extracellular environment [12], as major contributors to establishing the pathogenic state. Indeed, over the past 30 years or so, a small community of investigators has focused on the role of ion channels in cancer proliferation, metastasis and recurrence [13]. Voltage-gated Ca^2+^ and K^+^ channels, up-regulated in many forms of carcinoma and sarcoma, induce the suspension of apoptosis [13,14,15,16]. Treatments designed to inhibit Ca^2+^ channels can re-instate apoptosis [17,18]. Likewise, inhibition of voltage-gated K^+^ channels can restore apoptosis in many carcinomas [19]. Additionally, some ligand-gated ion channels, over-expressed in certain cancers, lead to enhanced cellular proliferation [20]. Importantly, an increase in voltage-gated sodium channel (VGSC) expression has been linked to increased motility, invasiveness, growth rate, and metastasis in most aggressive carcinomas [21,22,23,24,25,26,27,28,29,30,31,32]. Because of these findings, Prevarskaya et al. [33] proposed that cancer should viewed as an “oncochannelopathy” which has led to the proposal of an array of novel treatments that in deference to approaches that eliminate the affected cells to deliver a cure, are designed to additionally modulate or mitigate the effects of altered channel expression in order to restore function.

## 2. Ions and Ion Channels—Basic Fundamental of Life

### 2.1. Ion Channels and Life in Single Cells

Life as we know it is contingent upon the ability to isolate, organize, coordinate and maintain a conducive environment for chemical reactions to occur that transform or conserve energy to support growth, reproduction and survival of cellular units in an ever-changing and often hostile external environment. The types and relative amounts of charged elements present at a given time that can vary widely in the external environment, but are tightly controlled within the confines of individual cells by semi-permeable membranes that are responsible for maintaining cellular shape and the internal compartmentalization that is necessary for ensuring the proper and efficient conduction of biochemical reactions [34,35,36].

Neutral relationships in living systems fluctuate due to the semi-permeable nature of the plasma membrane and the ionic concentration gradients that provide a form of stored energy for driving many of life’s essential biologic reactions [37], e.g., neuronal action potential, muscular contraction, oxidative phosphorylation, in an effort to reach molecular equilibrium. The charged nature of the lipid bilayer also retains large, impermeable, negatively-charged, and osmotically active molecules within the cell that create a charge imbalance with the extracellular space. The charge imbalance must be reconciled in order to achieve osmotic equilibrium, homeostasis, and environmental support for biologic function and must be present to establish and maintain the voltage gradient across the membrane of active cells. Because the passage of each of the charged species differs, resolution of the charge imbalance must be achieved by coordinating the concentrations of charged species, typically Na^+^ ions with limited access to the intracellular space and less impeded, positively charged K^+^ ions. Based on the level of cellular activity and the composition of the extracellular space, the membrane potential can shift above or below the resting level, thus affecting conformational change in a variety of transmembrane proteins that selectively allow voltage-gated facilitated diffusion of specific charged species, e.g., K^+^, Ca^2+^, Na^+^, Mg^2+^, H^+^, Cl^−^, PO_4_^2−^ and HCO_3_^−^ ions, into and out of the intracellular space down their concentration gradients [38]. The gradients are created and maintained by the active transport of charge elements across cell membranes against their concentration gradient that derive energy from the breakdown of adenosine triphosphate (ATP) or by coupling the transport of charged particles against their concentration gradients in conjunction with the transfer of another charged element that flows down its concentration gradient [39,40]. The linked transport provides the requisite energy for the exchange, e.g., Na^+^/Ca^2+^ exchanger, Na^+^/H^+^ exchanger, Cl^−^/HCO_3_^−^ exchanger. Sodium-potassium-ATPase (Na^+^, K^+^-ATPase; the sodium pump), serves as the primary energy-dependent transporter in most cells for establishing and maintaining the electrochemical gradient that is created by the differences in the intra- and extracellular Na^+^ and K^+^ ion concentrations across the cell membrane [40]. Channel opening and closing and the ability to subsequently restore the membrane’s electrochemical gradient provides the basis for the cell’s ability to monitor the extracellular environment to embrace favorable and avoid injurious conditions and for maintaining proper cellular homeostasis for growth, cell motility and reproduction. Alterations in the voltage gradient that occur across plasma membranes in the process of living and in response to challenges from the extracellular environment result in a choreographed ebb and flow of charged elements that is necessary to adjust to changes that occur during the performance of biological functions. As long as individual cells are able to acquire oxygen and sufficient nutrients and to eliminate waste, they will grow and survive.

### 2.2. Ion Considerations in Multicellular Organisms

Ion concentrations and gradients and the modulation of the channels and transporters responsible for establishing and maintaining osmotic balance are also essential for survival at the level of multicellular organisms. The regulation of charge provides energy and establishes the basis for performing and maintaining many essential functions including the control of cellular replication, relative growth and cell death during embryonic development, the initiation of gene expression, organ function and plastic changes needed for organ repair and maintenance, the coordination of neuronal function, the development and modulation of neuronal circuitry controlling behavior and even the development of pathologic change associated with degenerative and proliferative disease that contributes to organismal demise [41].

Despite the significant specialization of function that is required to ensure the survival of multicellular organisms, the maintenance of cellular homeostasis and the elements of intercellular communication are conserved and are absolutely fundamental. They rely on the expression and coordinated functioning of ion channels to communicate and coordinate the benefits of cellular specialization to enable the organism to successfully compete for, and effectively occupy osmotically supportive niches for the good of the organism. In so doing, a conceptual shift in priority from ensuring survival of individual cells to survival of the whole is adopted. This requisite shift involves autophagy, a process for breaking down, eliminating or recycling damaged cellular components and the programmed elimination of certain cells to ensure optimum functioning of individual parts for the benefit of the whole, a process known as apoptosis or apoptotic cell death [42,43,44,45,46]. Autophagy serves to repair and replace dysfunctional cells and apoptosis plays an essential role in shaping an organism during embryonic development, in the maintenance of cellular volume and turnover and in the control of organ size.

**Figure 1:**
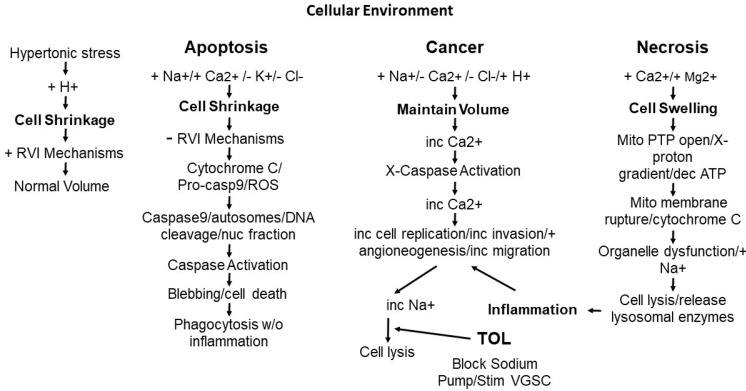
The diagram presents major steps in the metabolic pathways occur in cells in response to extrinsic environmental challenges (hypertonicity, hypoxia, toxins, immune response, and trauma) and intrinsic signaling that trigger influx (+) or efflux (−) of ions orchestrated through numerous channels between the extracellular matrix and the intracellular space resulting in shifts in cell volume, downstream activation, increase (inc), decrease (dec), or inhibition (X) of processes affecting cellular function and survival. The numbers and types of channels expressed and the resulting number of ions exchanged determines cellular functioning and provides targets for developing treatments to modulate pathologic change and provides the means for cells to develop resistance and avoid elimination with intervention affecting steps closer to cellular elimination having less opportunity for resistance.

As in life, the death of a cell depends on maintaining the proper water content within a space-limiting membrane. The free passage of water across the plasma membrane occurs by osmosis when differences in osmotic pressure exist between the cell’s external and internal environs [41,47,48,49]. In the presence of low osmotic pressure, water diffuses through the cell membrane resulting in an increase in cellular volume. Although water generally follows the flux of Na^+^, the restoration of cell volume in normal cells in response to small isovolumetric fluctuations in osmotic pressure, typically involves a large influx of Ca^2+^ and the extrusion of K^+^, Cl^−^, and organic osmolytes through activation of a wide variety of transmembrane protein channels in the cell membrane, e.g., Ca^2+^-activated K^+^ (K_Ca_) channels (predominantly the large-conductance (BK_Ca_) and intermediate-conductance (IK_Ca_) Ca^2+^-activated K^+^ channels), voltage-gated K^+^ (Kv) channels, inwardly rectifying K^+^ (K_IR_) channels, two-pore-domain K^+^ (K_2P_) channels and the extrusion of organic osmolytes, e.g., amino acids, polyalcohols, and amines, through volume-regulated anion channels (VRAC) associated with the production of free radicals [41,47,50,51]. In the presence of high osmotic pressure, water leaves the cell thereby reducing cell volume and initiating a regulatory volume increase (RVI) mechanism associated with increased levels of intracellular Na^+^, Cl^−^, and organic osmolytes through the activation of Na^+^/Cl^−^ and Na^+^/K^+^/2Cl cotransporters and Na^+^/H^+^ exchangers [52,53,54,55,56]; Figure 1. The maintenance of intracellular and extracellular ionic balance, osmotic pressure and cell volume, is essential for the support of life sustaining biochemical reactions and survival [42,43,47,55,57,58,59]. The mechanisms and elements enabling ionic exchange are conserved and extraordinarily redundant in cells throughout the animal kingdom [60,61]. When working well, the multiplicity of pathways effectively guarantees continued conduction of essential functions and provides a means to escape elimination when exposure to harmful or lethal stresses occurs.

### 2.3. Ion Channels, Cell Volume and Cell Death

**Figure 2:**
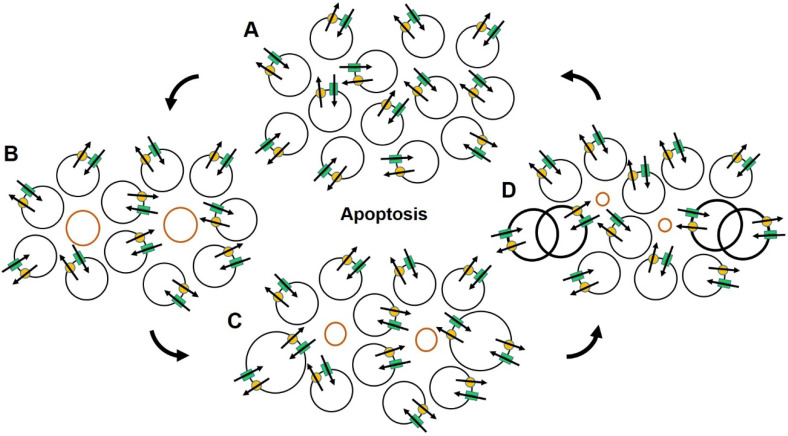
The illustration depicts important characteristics of normal apoptosis that rely on proper ion channel expression and function and are essential for maintaining healthy organ structure and integrity. The circular profiles represent relative size of normal cells (black circles) and cells scheduled for programmed cell death (red circles). Parallel green bars and yellow dots represent typical ion channels and their restorative mechanisms, here for Na^+^, respectively. Arrows denote the direction of ionic flow. Dividing cells are indicated by overlapping circles and bold lines. Beginning in (**A**), normal cells for a portion of functioning organ are rendered with relatively uniform size. As apoptosis begins (**B**), there is a shift in ion channel function in the cells scheduled for apoptotic death (red circles) resulting in a decrease in volume that progresses through (**C**,**D**) as the process continues. This process provides room for replacement cells seen dividing in (**D**). Upon completion of cell division, the remains of the dead cells are removed by phagocytosis maintaining structural integrity in (**A**), while renewing component cells. Note that this process occurs without initiating inflammation.

Loss of the ability to control volume is a characteristic of cells that foreshadows their demise [41]. In multicellular organisms, cells in the tissues that regularly sustain physical stress, exposure to toxins or injury necessitating frequent repair and an increase demand for DNA replication, are eliminated by autophagy or are genetically programmed to undergo elimination through a form of apoptotic cell death [42,43,44,45,46]. The elimination of fatigued or impaired cells reduces the likelihood of propagating errors in transcription and translation associated with damage to the genetic code and makes room for replacement cells. The process of apoptosis is characterized by a loss in cell volume. The volumetric change is affected through the coordinated activity of several transmembrane ion channels in the outer plasma membranes and inner mitochondrial membranes that are responsible for regulating intracellular osmotic pressure and production of ATP and the failure to initiate the RVI mechanism [56]; Figure 1. The process is set in motion by an increase in intracellular Na^+^ [62,63,64,65] that initiates an efflux of intracellular K^+^ through Kv, BK_Ca_, IK_Ca_, delayed rectifier and inward rectifier K^+^ channels, a process that blocks the apoptotic death receptor and reduces the protection against DNA fragmentation and caspase-3 protease activation [41,57,66,67,68,69,70]. The additional activation of volume sensitive, outwardly rectifying Cl^−^ channels [71,72,73,74,75] and Bax-associated inhibition of mitochondrial Kv 1.3 channels, further enables the release of cytochrome c and the production of reactive oxygen species that lead to apoptosis, the normal and evolutionarily beneficial cascade of events that encompasses cleavage and condensation of the DNA, nuclear fragmentation, caspase activation, apoptosome formation and apoptotic nuclease activity, resulting in blebbing of the plasma membrane, cell death, and engulfment of the cell by phagocytes [41,55,58,69,70,73,75,76,77,78,79,80,81,82,83,84,85]. These final steps remove non-functional cells without initiating inflammation and changes in the extracellular matrix associated with the requisite release of lytic enzymes and chemical mediators associated with cell death. The surviving cells’ avoidance of exposure to the deleterious effects of lytic inflammatory enzymes and chemical mediators associated with inflammation minimizes collateral damage in the surrounding extracellular environment and maximally preserves organ function and integrity (Figure 2).

**Figure 3:**
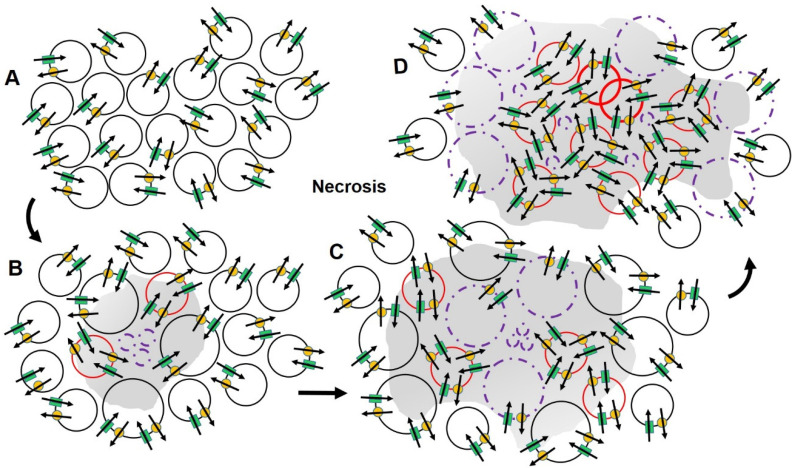
The illustration presents important characteristics of necrosis that distinguish this form of cell death with apoptosis. Conventions are the same as used in Figure 1. There are normal cells in (**A**). A nidus of injury (small purple circles) and an area of inflammation (shaded area) initiated to control the amount of injury and heal the wound is illustrated in (**B**). The inflammatory mediators affect a change in channel expression in local cells resulting in an increase in cell volume for normal cells and a maintenance of cell volume in cells scheduled for apoptotic death. In (**C**), lytic enzymes in the area of inflammation affect lethal changes in the genetic code and initiate necrosis in the previously normal cells (large circles with dashed purple borders). These changes lead to the extension of the area of inflammation and enhance the effect on channel expression and function. Shifts in channel expression and function in response to changes in the extracellular matrix illustrated in (**D**), support the cells scheduled for apoptosis channel. Increases in Na^+^ channel expression supports survival and enhances cell motility and the ability to invade to areas beyond the area of inflammation.

By contrast, necrotic cell death stems from pathogenic attack, ischemia, or the irreversible thermal, mechanical or chemical compromise of cell structure, or the mechanisms needed to store and use energy for cellular functioning, is associated with cellular swelling. Necrotic volume increases involve increased Ca^2+^ influx through activation of members of the family of transient receptor potential (TRP) channels, disruption of chromatin and ion and electron transport mechanisms that lead to a depletion or reduced synthesis of ATP, organelle dysfunction, the influx of Na^+^ through TRP melastatin-2 and -4 channels, and cell lysis [47,81,82]; Figure 1. With disruption in the cell membrane, lysosomal enzymes and chemical mediators are released into the surrounding extracellular matrix initiating the process of inflammation and the recruitment of components of the immune system to restrict the extent of damage, remove the cause and result of injury, repair structural integrity and restore normal homeostasis and function [86]; Figure 3. This process generally produces sufficient, but often imperfect results that can ultimately lead to a reduction in cellular reserve, structural deterioration, and loss of function. This process can accelerate in the presence of especially harsh environments associated with frequent or extensive physical or hypoxic stress or exposure to toxic/metabolic cellular trauma and tissue injury (Figure 1). Prolonged periods of inflammation with its requisite release of chemokines, growth factors and proteolytic enzymes, can increase the risk of producing collateral damage in normal tissue [87,88]. Chronic inflammation thus hastens the rate of decline associated with wear and tear and imposes an increased need for repair and replacement of essential cellular constituents that are crucial for maintaining function and survival. Coincidentally, the release of chemical mediators and growth factors associated with the cyclooxygenase pathway that are released during inflammation and are known to stimulate the expression of VGSCs in dorsal root ganglia related to the site of inflammation [89,90,91]. It may similarly affect cells that surround an area of necrosis by increasing the expression of VGSCs, osmotic influx of water and cell swelling. The addition of regulated conduits for Na^+^ influx may provide a means for preempting the apoptotic reduction in cell volume and programmed cell death, resulting in prolongation of cell survival and an enhanced ability for the affected cells to metastasize and invade normal tissue [92,93,94].

### 2.4. Ion Channels and Cancer

With age, time and each replication associated with frequent exposure to necrosis and inflammation, the likelihood for mutation of the genetic code increases and the cell’s ability to repair the damage declines. Alterations associated with ion channel expression play a role in altering apoptotic balance that leads to increased cellular longevity and an enhanced ability for cellular proliferation [95,96,97]. For example, changes in the expression of key membrane channels or transporters affect change through modulation of the pathways for ion exchange that regulate energy utilization and storage necessary to support the performance of the biological reactions that are critical for maintaining cell volume, osmotic balance and survival of cancer cells. Following oncogenic change, neoplastic cells increase water content and maintain cell volume by decreasing K^+^ efflux, at least in part by reducing the expression of a number of cell type- and state-specific, Kv, including among others Kv 1.1, Kv 1.3, Kv 1.5, Kv 2.1 and Kv 11.1 [75,76,98,99,100,101,102,103,104,105], by increasing the expression of VGSCs [21,22,23,24,25,26,27,28,29,30,31,32,106], Ca^2+^-activated Cl^−^ channels, 8-transmembrane receptor-activated Cl^−^ channels, and volume sensitive outwardly rectifying Cl^−^ channels [107,108,109]. Additionally, alterations in the microenvironment further support the invasion of transformed cells into normal tissue and promote their growth and metastasis [110,111].

Because of the recognized importance of ions, ion channels and transporter expression in establishing, maintaining and restoring ion balance, homeostasis and essential concentration gradients involved in the regulation of the cell cycle [112], cell growth [95,113], proliferation [96,100,103,113], migration [97,98], neovascularization [114], and the cascades of events related to programmed cell death [115,116], considerable effort has been devoted to the discovery and development of therapeutic agents that can be targeted to affect changes, particularly in K^+^, Na^+^ and Cl^−^ channel function or expression that make it possible for cells in specific degenerative or neoplastic disease states to accelerate or evade programmed cell death. In the case of cancer, these cells additionally invade normal tissue and metastasis [21,22,24,25,92,93,94,95,99,100,101,111,113]; Figure 1. Unfortunately, because of the rapid growth of tumors, cellular microenvironments continually evolve and generate novel challenges for cancer cells to meet and overcome in order to survive. The multiplicity of ion channels and redundancy of options for ion-ion interactions provides virtually endless possibilities for compensatory downstream ‘by-pass’ alternatives for evading destruction when preferred paths are compromised [114]; Figure 1. This confers enhanced survival of the cancer and a monumental challenge for the clinician wishing to select an appropriate target for achieving therapeutic benefit while averting treatment failure and adverse events.

Initial attempts at developing treatments were focused on altered ion channel expression involved in the initiation or reversal of the pathologic changes in the apoptotic cascades, the ability of cells to reproduce, or the development of resistance to treatment. These technologies logically employed the promising and rapidly expanding approaches of targeted and immune-modulating therapies that are designed to identify features unique to diseased cells that can be used to deliver therapeutic agents or activate and direct immune attacks to eliminate the pathologic cells and are the mainstay of our current standard of care [111,112,117,118,119,120]. While the selectivity achieved by activation or inhibition of gene expression and targeting the delivery of therapeutic interventions has improved efficacy and limited adverse effects compared with earlier therapeutic methods, that they affect permanent change or lethality in targeted cells, encumbers these treatments with significant adverse effects related to cross-reactions with a wide variety of conserved ion channels that exist and are broadly expressed in normal tissues. For example, pharmacological or pathologic inhibition of production of Nav 1.5 channels that are over-expressed in many carcinomas can produce cardiomyopathies, cardiac arrhythmias, dizziness and nausea [121].

As in other channelopathies, unintended problems associated with targeting and eliminating specific abnormally expressed ion channels and the cells in which they are commonly expressed is acknowledged to be especially problematic when considering irreversible treatments have the potential to eliminate all cells that normally or abnormally express the targeted channel, effectively compromising or destroying organ systems, e.g., cardiac, peripheral nerve, and precluding survival. For example, agents designed to directly target and reduce the overexpression of Kv, especially the Kv 1.3 and Kv 1.5 channels, or alter the expression of proteins, such as K^+^ channel modulatory protein KChAP [105] or Bax, a pro-apoptotic protein [43,76,77], that through interaction with K^+^ ion channels in the mitochondrial membrane compromise of the oxidative phosphorylation cascade may, by providing a mechanism to alter intracellular concentration gradients, decrease neoplastic resistance and stimulate apoptosis, but may also lead to cardiac arrhythmias.

The features of phenotypic heterogeneity and genetic heterogeneity (mutations in a single gene that can cause different diseases and mutations in different genes that can result in the same or similar disease phenotype), impose additional complications for identifying and delivering optimum treatment. For example, mibefradil, a Ca^2+^ channel inhibitor, inhibits proliferation in human glioma and neuroblastoma cells, but because of its effect on the functioning in off-target epithelial cells may have the opposite effect through modulation of Ca^2+^-activated Cl^−^ channels that have been reported to stimulate apoptosis and suppress tumour formation [107].

Treatments of channelopathies, are therefore conceptually different from other treatments because they focus on developing reversible methods to directly or indirectly modulate ion balance and channel activity in abnormally expressing cells to achieve control of the disease through reversal of the gain or loss of channel function. In the management of cancer, as in the management of inherited painful channelopathies, the use of Na^+^ channel blocking agents, such as carbamazepine, phenytoin and riluzole, that that block the expression or impede the function of VGSCs has been shown to decrease tumor growth and invasiveness and to suppress cell migration and metastasis, a noble aim. However, as with other agents that target the elimination of functional gain, have thus far been ineffective in eliminating the cancer [39,92,122,123,124,125,126,127,128,129].

### 2.5. An Alternate Approach to Conventional Therapy

To minimize the likelihood of treatment failure and adverse effects, short-lived, ion channel blocking agents affecting reversible compromise of common path conduits that govern cellular homeostasis, osmotic pressure and cell volume, essential for survival with few, if any, escape options and greatest disparity between normal and diseased cells are likely to provide the best therapeutic targets (Figure 1). Evidence provided in recent reports on the benefits of ‘targeted osmotic lysis’ (TOL) support the possibility that Na^+^ channel activation and simultaneous blockade of Na^+^, K^+^-ATPase may offer a promising option for the treatment of advanced carcinoma because it affects a mechanism that is a final common path to survival with fewer options for evasion [93,94,118,128,129]. Three decades of evidence shows that most aggressive carcinomas overexpress VGSCs. This overexpression confers an enhanced ability to invade normal tissue and to metastasize [21,22,23,24,25,26,27,28,29,30,31,32]. The dynamic relationship between VGSCs and Na^+^, K^+^-ATPase is essential for maintaining the critical Na^+^/K^+^ electrochemical gradient and cellular homeostasis. The understanding of this relationship led to the hypothesis that because of the similar importance of the conserved relationship between Na^+^ channels and the Na^+^ pump, it might be possible to selectively eliminate many carcinomas that highly over express VGSCs by augmenting, rather than blocking, VGSC activity and simultaneously blocking the pumping mechanism responsible for restoring the Na^+^/K^+^ electrochemical gradient and cellular homeostasis [94,95].

In TOL, opening of VGSCs is achieved by delivering a pulsed electric field to the whole body. Because of the relative negativity of the intracellular environment and extracellular Na^+^ levels always exceed those in the intracellular space, influx of Na^+^ into the overly expressing cancer cells greatly increases down this concentration gradient, usually about 30:1. The simultaneous blocking the Na^+^ pumping mechanism with a cardiac glycoside prevents the return of the ions to the extracellular space. Water then passively follows Na^+^ into the cells by osmosis, in an attempt to restore normal oncotic pressure, but exceeding the cells capacity to comply, resulting in cell lysis. By contrast, normal cells are spared from damage because Na^+^ channel expression is significantly less than that found in most advanced carcinomas. Thus, less Na^+^, and consequently less water, enters normal cells averting significant cell swelling and lysis. The evidence to date, drawn from studies conducted on several forms of cancer performed in vitro and in vivo in several mammalian species supports the approach to treatment and that the augmentation of abnormality, particularly with reference to TOL has the potential to provide a safe, well-tolerated and effective treatment for advanced carcinomas without compromising quality of life [92,93,118,130,131].

## 3. Conclusions

Because of their ubiquitous distribution, conserved nature and functional characteristics in all living cells, ions and the transmembrane channels and transporters that determine and maintain the relationships that are the essence of life and survival, and the recognition that unique shifts in channel expression and activity occur and serve in the pathogenesis of disease, the modulation of charge offers an attractive target for developing new and effective therapies for managing disease. Importantly, because of the evidence of efficacy for TOL that has been presented to date for the treatment of advanced carcinoma, we further propose that the identification of shifts in ion channel expression that are widely disparate from that observed in normal cells, play an integral part of ion-ion relationships critical to survival, and are characteristic, causative and unique to the pathogenesis of disease, may prove to be and should be considered in developing novel and effective methods of treatment. In addition, the augmentation of altered modulating determinants alone or in combination with complementary therapies warrants consideration and evaluation as they might well add a new dimension to our approach to managing neoplastic and other debilitating diseases that may be as good or more effective than standard approaches designed to impede or eliminate the inciting cause.

## 4. Patents

A patent for the technology described in this manuscript entitled, Targeted Osmotic Lysis of Cancer Cells—File No. 11M01 (Serial No. 13/552,909) Paul DJ and Gould HJ III was allowed on 30 December 2014.

## References

[1] Ptáček L., George A.L., Griggs R.C., Tawil R., Kallen R.G., Barchi R.L., Robertson M., Leppert M.F. (1991). Identification of a mutation in the gene causing hyperkalemic periodic paralysis. Cell.

[2] Ptáček L.J., Trimmer J.S., Agnew W.S., Roberts J.W., Petajan J.H., Leppert M. (1991). Paramyotonia congenita and hyperkalemic periodic paralysis map to the same sodium-channel gene locus. Am. J. Hum. Genet..

[3] Ptáček L.J., Tyler F., Trimmer J.S., Agnew W.S., Leppert M. (1991). Analysis in a large hyperkalemic periodic paralysis pedigree supports tight linkage to a sodium channel locus. Am. J. Hum. Genet..

[4] Griggs R.C., Nutt J.G. (1995). Episodic ataxias as channelopathies. Ann. Neurol..

[5] Kullmann D.M. (2002). The neuronal channelopathies. Brain.

[6] Dib-Hajj S.D., Rush A.M., Cummins T.R., Hisama F.M., Novella S., Tyrrell L., Marshall L., Waxman S.G. (2005). Gain-of-function mutation in Nav1.7 in familial erythromelalgia induces bursting of sensory neurons. Brain.

[7] Cox J.J., Reimann F., Nicholas A.K., Thornton G., Roberts E., Springell K., Karbani G., Jafri H., Mannan J., Raashid Y. (2006). An SCN9A channelopathy causes congenital inability to experience pain. Nature.

[8] Drenth J.P.H., Waxman S.G. (2007). Mutations in sodium-channel gene SCN9A cause a spectrum of human genetic pain disorders. J. Clin. Investig..

[9] Kim J.-B. (2014). Channelopathies. Korean J. Pediatr..

[10] Schattling B., Eggert B., Friese M.A. (2014). Acquired channelopathies as contributors to development and progression of multiple sclerosis. Exp. Neurol..

[11] Musto E., Gardella E., Møller R.S. (2020). Recent advances in treatment of epilepsy-related sodium channelopathies. Eur. J. Paediatr. Neurol..

[12] Bernard G., Shevell M.I. (2008). Channelopathies: A review. Pediatr. Neurol..

[13] Litan A., Langhans S.A. (2015). Cancer as a channelopathy: Ion channels and pumps in tumor development and progression. Front. Cell. Neurosci..

[14] Huang X., Jan L.Y. (2014). Targeting potassium channels in cancer. J. Cell Biol..

[15] Anderson K.J., Cormier R.T., Scott P.M. (2019). Role of ion channels in gastrointestinal cancer. World J. Gastroenterol..

[16] Zhang L., Bing S., Dong M., Lu X., Xiong Y. (2021). Targeting ion channels for the treatment of lung cancer. Biochim. Biophys. Acta Rev. Cancer.

[17] Bulk E., Todesca L.M., Schwab A. (2021). Ion Channels in Lung Cancer. Rev. Physiol. Biochem. Pharmacol..

[18] Takayasu T., Kurisu K., Esquenazi Y., Ballester L.Y. (2020). Ion Channels and Their Role in the Pathophysiology of Gliomas. Mol. Cancer Ther..

[19] Rodat-Despoix L., Chamlali M., Ouadid-Ahidouch H. (2021). Ion channels as key partners of cytoskeleton in cancer disease. Biochim. Biophys. Acta Rev. Cancer.

[20] Bates E. (2015). Ion channels in development and cancer. Annu. Rev. Cell Dev. Biol..

[21] Fraser S.P., Salvador V., Manning E.A., Mizal J., Altun S., Raza M., Berridge R.J., Djamgoz M.B.A. (2003). Contribution of functional voltage-gated Na^+^ channel expression to cell behaviors involved in the metastatic cascade in rat prostate cancer: I. Lateral motility. J. Cell. Physiol..

[22] Fraser S.P., Diss J.K.J., Chioni A.M., Mycielska M.E., Pan H., Yamaci R.F., Pani F., Siwy Z., Krasowska M., Grzywna Z. (2005). Voltage-gated sodium channel expression and potentiation of human breast cancer metastasis. Clin. Cancer Res..

[23] Onkal R., Djamgoz M.B. (2009). Molecular pharmacology of voltage-gated sodium channel expression in metastatic disease: Clinical potential of neonatal Na_V_1.5 in breast cancer. Eur. J. Pharmacol..

[24] Bennett E.S., Smith B.A., Harper J.M. (2004). Voltage-gated Na^+^ channels confer invasive properties on human prostate cancer cells. Pflüg. Arch..

[25] Brackenbury W.J., Chioni A.M., Diss J.K., Djamgoz M.B.A. (2007). The neonatal splice variant of Nav1.5 potentiates in vitro invasive behaviour of MDA-MB-231 human breast cancer cells. Breast Cancer Res. Treat..

[26] Gao R., Shen Y., Cai J., Lei M., Wang Z. (2010). Expression of voltage-gated sodium channel alpha subunit in human ovarian cancer. Oncol. Rep..

[27] Gillet L., Roger S., Besson P., Lecaille F., Gore J., Bougnoux P., Lalmanach G., Le Guennec J.-Y. (2009). Voltage-gated sodium channel activity promotes cysteine cathepsin-dependent invasiveness and colony growth of human cancer cells. J. Biol. Chem..

[28] Grimes J.A., Fraser S.P., Stephens G.J., Downing J.E., Laniado M.E., Foster C.S., Abel P.D., Djamgoz M.B. (1995). Differential expression of voltage-activated Na^+^ currents in two prostatic tumour cell lines: Contribution to invasiveness in vitro. FEBS Lett..

[29] House C.D., Vaske C.J., Schwartz A.M., Obias V., Frank B., Luu T., Sarvazyan N., Irby R., Strausberg R.L., Hales T.G. (2010). Voltage-gated Na^+^ channel SCN5A is a key regulator of a gene transcriptional network that controls colon cancer invasion. Cancer Res..

[30] Onganer P.U., Djamgoz M.B. (2005). Small-cell lung cancer (human): Potentiation of endocytic membrane activity by voltage-gated Na(+) channel expression in vitro. J. Membr. Biol..

[31] Fraser S.P., Ozerlat-Gunduz I., Brackenbury W.J., Fitzgerald E.M., Campbell T.M., Coombes R.C., Djamgoz M.B. (2014). Regulation of voltage-gated sodium channel expression in cancer: Hormones, growth factors and auto-regulation. Philos. Trans. R. Soc. Lond. B Biol. Sci..

[32] Roger S., Rollin J., Barascu A., Besson P., Raynal P.-I., Iochmann S., Lei M., Bougnoux P., Gruel Y., Le Guennec J.-Y. (2007). Voltage-gated sodium channels potentiate the invasive capacities of human non-small-cell lung cancer cell lines. Int. J. Biochem. Cell Biol..

[33] Prevarskaya N., Skryma R., Shuba Y. (2018). Ion Channels in Cancer: Are Cancer Hallmarks Oncochannelopathies?. Physiol. Rev..

[34] Bailey R. (2020). Cell Membrane Function and Structure. ThoughtCo.

[35] Marshall W.F., Young K.D., Swaffer M., Wood E., Nurse P., Kimura A., Frankel J., Wallingford J., Walbot V., Qu X. (2012). What determines cell size?. BMC Biol..

[36] Krapf D. (2018). Compartmentalization of the plasma membrane. Curr. Opin. Cell Biol..

[37] Deamer D., Gargaud M., Amils R., Quintanilla J.C., Cleaves H.J., Irvine W.M., Pinti D.L., Viso M. (2011). Concentration Gradients. Encyclopedia of Astrobiology.

[38] Barros L.F., Castro J., Bittner C.X. (2002). Ion movements in cell death: From protection to execution. Biol. Res..

[39] Lang F., Christos Stournaras C. (2014). Ion channels in cancer: Future perspectives and clinical potential. Philos. Trans. R. Soc. Lond. B Biol. Sci..

[40] Kumari J., Rathore M.S. (2020). Na^+^/K^+^-ATPase a Primary Membrane Transporter: An Overview and Recent Advances with Special Reference to Algae. J. Membr. Biol..

[41] Dubois J.-M., Rouzaire-Dubois B. (2012). Roles of cell volume in molecular and cellular biology. Prog. Biophys. Mol. Biol..

[42] Hoffmann E.K., Lambert I.H., Pedersen S.F. (2009). Physiology of cell volume regulation in vertebrates. Physiol. Rev..

[43] Lambert I.H., Hoffmann E.K., Pedersen S.F. (2008). Cell volume regulation: Physiology and pathophysiology. Acta Physiol..

[44] Kondratskyi A., Kondratska K., Skryma R., Prevarskaya N. (2015). Ion channels in the regulation of apoptosis. Biochim. Biophys. Acta.

[45] Oberst A., Bender C., Green D.R. (2008). Living with death: The evolution of the mitochondrial pathway of apoptosis in animals. Cell Death Differ..

[46] Kondratskyi A., Kondratska K., Skryma R., Klionsky D.J., Prevarskaya N. (2018). Ion channels in the regulation of autophagy. Autophagy.

[47] McFerrin M.B., Turner K.L., Cuddapah V.A., Sontheimer H. (2012). Differential role of IK and BK potassium channels as mediators of intrinsic and extrinsic apoptotic cell death. Am. J. Physiol. Cell Physiol..

[48] Pasantes-Morales H. (2016). Channels and volume changes in the life and death of the cell. Mol. Pharmacol..

[49] Eveloff J.L., Warnock D.G. (1987). Activation of ion transport systems during cell volume regulation. Am. J. Physiol..

[50] Di Meo S., Reed T.T., Venditti P., Victor V.M. (2016). Role of ROS and RNS sources in physiological and pathological conditions. Oxidative Med. Cell. Longev..

[51] Friard J., Laurain A., Rubera I., Duranton C. (2021). LRRC8/VRAC channels and the redox balance: A complex relationship. Cell Physiol. Biochem..

[52] Božič B., Jokhadar Š.Z., Kristanc L., Gregor Gomišček G. (2020). Cell volume changes and membrane ruptures induced by hypotonic electrolyte and sugar solutions. Front. Physiol..

[53] Alexander R.T., Grinstein S. (2006). Na^+^/H^+^ exchangers and the regulation of volume. Acta Physiol..

[54] Burg M.B., Ferraris J.D., Dmitrieva N.I. (2007). Cellular response to hyperosmotic stresses. Physiol. Rev..

[55] Arroyo J.P., Kahle K.T., Gamba G. (2013). The SLC12 family of electroneutral cation-coupled chloride cotransporters. Mol. Asp. Med..

[56] Maeno E., Takahashi N., Okada Y. (2006). Dysfunction of regulatory volume increase is a key component of apoptosis. FEBS Lett..

[57] Cala P.M. (1980). Volume regulation by Amphiuma red blood cells. The membrane potential and its implications regarding the nature of the ion-flux pathways. J. Gen. Physiol..

[58] Lang F., Föller M., Lang K.S., Lang P.A., Ritter M., Gulbins E., Vereninov A., Huber S.M. (2005). Ion channels in cell proliferation and apoptotic cell death. J. Membr. Biol..

[59] Stutzin A., Hoffmann E.K. (2006). Swelling-activated ion channels: Functional regulation in cell-swelling, proliferation and apoptosis. Acta Physiol..

[60] Cai X. (2012). Evolutionary genomics reveals the premetazoan origin of opposite gating polarity in animal-type voltage-gated ion channels. Genomics.

[61] Murthy S.E., Dubin A.E., Whitwam T., Jojoa-Cruz S., Cahalan S.M., Mousavi S.A.R., Ward A.B., Patapoutian A. (2018). OSCA/TMEM63 are an Evolutionarily Conserved Family of Mechanically Activated Ion Channels. eLife.

[62] Riedl S.J., Salvesen G.S. (2007). The apoptosome: Signalling platform of cell death. Nat. Rev. Mol. Cell Biol..

[63] Bao Q., Shi Y. (2007). Apoptosome: A platform for the activation of initiator caspases. Cell Death Differ..

[64] Franco R., Bortner C.D., Cidlowski J.A. (2006). Potential roles of electrogenic ion transport and plasma membrane depolarization in apoptosis. J. Membr. Biol..

[65] Bortner C.D., Sifre M.I., Cidlowski J.A. (2008). Cationic gradient reversal and cytoskeleton-independent volume regulatory pathways define an early stage of apoptosis. J. Biol. Chem..

[66] Bortner C.D., Cidlowski J.A. (2003). Uncoupling cell shrinkage from apoptosis reveals that Na^+^ influx is required for volume loss during programmed cell death. J. Biol. Chem..

[67] Yurinskaya V., Goryachaya T., Guzhova I., Moshkov A., Rozanov Y., Sakuta G., Shirokova A., Shumilina E., Vassilieva I., Lang F. (2005). Potassium and sodium balance in U937 cells during apoptosis with and without cell shrinkage. Cell. Physiol. Biochem..

[68] Bortner C.D., Cidlowski J.A. (2007). Cell shrinkage and monovalent cation fluxes: Role in apoptosis. Arch. Biochem. Biophys..

[69] Hughes F.M., Bortner C.D., Purdy G.D., Cidlowski J.A. (1997). Intracellular K^+^ suppresses the activation of apoptosis in lymphocytes. J. Biol. Chem..

[70] Thompson G.J., Langlais C., Cain K., Conley E.C., Cohen G.M. (2001). Elevated extracellular [K^+^] inhibits death-receptor- and chemical-mediated apoptosis prior to caspase activation and cytochrome c release. Biochem. J..

[71] Bortner C.D., Cidlowski J.A. (2020). Ions, the movement of water and the apoptotic volume decrease. Front. Cell. Dev. Biol..

[72] Lang F., Föller M., Lang K., Lang P., Ritter M., Vereninov A., Szabo I., Huber S.M., Gulbins E. (2007). Cell volume regulatory ion channels in cell proliferation and cell death. Methods Enzymol..

[73] Okada Y., Shimizu T., Maeno E., Tanabe S., Wang X., Takahashi N. (2006). Volume-sensitive chloride channels involved in apoptotic volume decrease and cell death. J. Membr. Biol..

[74] Wang X., Takahashi N., Uramoto H., Okada Y. (2005). Chloride channel inhibition prevents ROS-dependent apoptosis induced by ischemia-reperfusion in mouse cardiomyocytes. Cell. Physiol. Biochem..

[75] Okada Y., Maeno E., Shimizu T., Manabe K., Mori S.-I., Nabekura T. (2004). Dual roles of plasmalemmal chloride channels in induction of cell death. Pflug. Arch..

[76] Szabó I., Bock J., Grassmé H., Soddemann M., Wilker B., Lang F., Zoratti M., Gulbins E. (2008). Mitochondrial potassium channel Kv1.3 mediates Bax-induced apoptosis in lymphocytes. Proc. Natl. Acad. Sci. USA.

[77] Leanza L., Zoratti M., Gulbins E., Szabò I. (2012). Induction of apoptosis in macrophages via Kv1.3 and Kv1.5 potassium channels. Curr. Med. Chem..

[78] Cain K., Langlais C., Sun X.M., Brown D.G., Cohen G.M. (2001). Physiological concentrations of K^+^ inhibit cytochrome c-dependent formation of the apoptosome. J. Biol. Chem..

[79] Bortner C.D., Hughes F.M., Cidlowski J.A. (1997). A primary role for K^+^ and Na^+^ efflux in the activation of apoptosis. J. Biol. Chem..

[80] Bortner C.D., Cidlowski J.A. (2011). Life and death of lymphocytes: A volume regulation affair. Cell. Physiol. Biochem..

[81] Ferrera L., Barbieri R., Picco C., Zuccolini P., Remigante A., Bertelli S., Fumagalli M.R., Zifarelli G., Porta C.A.M., Gavazzo P. (2021). TRPM2 oxidation activates two distinct potassium channels in melanoma cells through intracellular calcium increase. Int. J. Mol. Sci..

[82] Girault A., Ahidouch A., Ouadid-Ahidouch H. (2020). Roles for Ca^2+^ and K^+^ channels in cancer cells exposed to the hypoxic tumour microenvironment. Biochim. Biophys. Acta Mol. Cell Res..

[83] Maeno E., Ishizaki Y., Kanaseki T., Hazama A., Okada Y. (2000). Normotonic cell shrinkage because of disordered volume regulation is an early prerequisite to apoptosis. Proc. Natl. Acad. Sci. USA.

[84] Schwartzman R.A., Cidlowski J.A. (1993). Apoptosis: The biochemistry and molecular biology of programmed cell death. Endocr. Rev..

[85] Bortner C.D., Cidlowski J.A. (2014). Ion channels and apoptosis in cancer. Philos. Trans. R. Soc. Lond. B Biol. Sci..

[86] Kerr J.F., Searle J. (1972). A suggested explanation for the paradoxically slow growth rate of basal-cell carcinomas that contain numerous mitotic figures. J. Pathol..

[87] Kroemer G., Galluzzi L., Vandenabeele P., Abrams J., Alnemri E.S., Baehrecke E.H., Blagosklonny M.V., El-Deiry W.S., Golstein P., Green D.R. (2009). Classification of cell death: Recommendations of the Nomenclature Committee on Cell Death 2009. Cell Death Differ..

[88] Rock K.L., Kono H. (2008). The inflammatory response to cell death. Annu. Rev. Pathol..

[89] Gould H.J., England J.D., Liu Z.P., Levinson S.R. (1998). Rapid sodium channel augmentation in response to inflammation induced by complete Freund’s adjuvant. Brain Res..

[90] Gould H.J., Gould T.N., England J.D., Paul D., Liu Z.P., Levinson S.R. (2000). A possible role for nerve growth factor in the augmentation of sodium channels in models of chronic pain. Brain Res..

[91] Gould H.J., England J.D., Soignier R.D., Nolan P., Minor L.D., Liu Z.P., Levinson S.R., Paul D. (2004). Ibuprofen blocks changes in Na v 1.7 and 1.8 sodium channels associated with complete Freund’s adjuvant-induced inflammation in rat. J. Pain.

[92] Li M., Xiong Z.-G. (2011). Ion channels as targets for cancer therapy. Int. J. Physiol. Pathophysiol. Pharmacol..

[93] Gould H.J., Norleans J., Ward T.D., Reid C., Paul D. (2018). Selective lysis of breast carcinomas by simultaneous stimulation of sodium channels and blockade of sodium pumps. Oncotarget.

[94] Paul D., Maggi P., Piero F.D., Scahill S.D., Sherman K.J., Edenfield S., Gould H.J. (2020). Targeted osmotic lysis of highly invasive breast carcinomas using a pulsed magnetic field and pharmacological blockade of voltage-gated sodium channels. Cancers.

[95] Lang F., Gulbins E., Szabo I., Lepple-Wienhues A., Huber S.M., Duranton C., Lang K.S., Lang P.A., Wieder T. (2004). Cell volume and the regulation of apoptotic cell death. J. Mol. Recognit..

[96] Lang F., Shumilina E., Ritter M., Gulbins E., Vereninov A., Huber S.M. (2006). Ion channels and cell volume in regulation of cell proliferation and apoptotic cell death. Contrib. Nephrol..

[97] Vandenberg C.A. (2008). Integrins step up the pace of cell migration through polyamines and potassium channels. Proc. Natl. Acad. Sci. USA.

[98] Szabò I., Zoratti M., Gulbins E. (2010). Contribution of voltage-gated potassium channels to the regulation of apoptosis. FEBS Lett..

[99] Pardo L.A. (2004). Voltage-gated potassium channels in cell proliferation. Physiology.

[100] Kaczmarek L.K. (2006). Non-conducting functions of voltage-gated ion channels. Nat. Rev. Neurosci..

[101] Felipe A., Bielanska J., Comes N., Vallejo A., Roig S., Ramón Y., Cajal S., Condom E., Hernández-Losa J., Ferreres J.C. (2012). Targeting the voltage-dependent K(+) channels Kv1.3 and Kv1.5 as tumor biomarkers for cancer detection and prevention. Curr. Med. Chem..

[102] Duvvuri U., Shiwarski D.J., Xiao D., Bertrand C., Huang X., Edinger R.S., Rock J.R., Harfe B.D., Henson B.J., Kunzelmann K. (2012). TMEM16A induces MAPK and contributes directly to tumorigenesis and cancer progression. Cancer Res..

[103] Spitzner M., Ousingsawat J., Scheidt K., Kunzelmann K., Schreiber R. (2007). Voltage-gated K^+^ channels support proliferation of colonic carcinoma cells. FASEB J..

[104] Kessler W., Budde T., Gekle M., Fabian A., Schwab A. (2008). Activation of cell migration with fibroblast growth factor-2 requires calcium-sensitive potassium channels. Pflüg. Arch..

[105] Wible B.A., Wang L., Kuryshev Y.A., Basu A., Haldar S., Brown A.M. (2002). Increased K^+^ efflux and apoptosis induced by the potassium channel modulatory protein KChAP/PIAS3beta in prostate cancer cells. J. Biol. Chem..

[106] Roger S., Potier M., Vandier C., Besson P., Le Guennec J.-Y. (2006). Voltage-gated sodium channels: New targets in cancer therapy?. Curr. Pharm. Des..

[107] Elble R.C., Pauli B.U. (2001). Tumor suppression by a proapoptotic calcium-activated chloride channel in mammary epithelium. J. Biol. Chem..

[108] Shimizu T., Numata T., Okada Y. (2004). A role of reactive oxygen species in apoptotic activation of volume-sensitive Cl(−) channel. Proc. Natl. Acad. Sci. USA.

[109] Spitzner M., Martins J.R., Soria R.B., Ousingsawat J., Scheidt K., Schreiber S., Kunzelmann K. (2008). Eag1 and Bestrophin 1 are up-regulated in fast-growing colonic cancer cells. J. Biol. Chem..

[110] Iorio J., Petroni G., Duranti C., Lastraioli E. (2019). Potassium and Sodium Channels and the Warburg Effect: Biophysical Regulation of Cancer Metabolism. Bioelectricity.

[111] Leslie T.K., James A.D., Zaccagna F., Grist J.T., Deen S., Kennerley A., Riemer F., Kaggie J.D., Gallagher F.A., Gilbert F.J. (2019). Sodium homeostasis in the tumour microenvironment. Biochim. Biophys. Acta Rev. Cancer.

[112] Lehen’kyi V., Shapovalov G., Skryma R., Prevarskaya N. (2011). Ion channels and transporters in cancer. 5. Ion channels in control of cancer and cell apoptosis. Am. J. Physiol. Cell Physiol..

[113] Pedersen S.F., Stock C. (2013). Ion channels and transporters in cancer: Pathophysiology, regulation, and clinical potential. Cancer Res..

[114] Negri S., Faris P., Berra-Romani R., Guerra G., Moccia F. (2020). Endothelial transient receptor potential channels and vascular remodeling: Extracellular Ca^2+^ entry for angiogenesis, arteriogenesis and vasculogenesis. Front. Physiol..

[115] Kunzelmann K. (2005). Ion channels and cancer. J. Membr. Biol..

[116] Bortner C.D., Cidlowski J.A. (2002). Cellular mechanisms for the repression of apoptosis. Annu. Rev. Pharmacol. Toxicol..

[117] Yang M., Kozminski D.J., Wold L.A., Modak R., Calhoun J.D., Isom L.L., Brackenbury W.J. (2012). Therapeutic potential for phenytoin: Targeting Na(v)1.5 sodium channels to reduce migration and invasion in metastatic breast cancer. Breast Cancer Res. Treat..

[118] Gould H.J., Paul D. (2022). Targeted osmotic lysis: A novel approach to targeted cancer therapies. Biomedicines.

[119] Hanahan D., Weinberg R.A. (2000). The hallmarks of cancer. Cell.

[120] Remigante A., Zuccolini P., Barbieri R., Ferrera L., Morabito R., Gavazzo P., Pusch M., Picco C. (2021). NS-11021 modulates cancer-associated processes independently of BK channels in melanoma and pancreatic duct adenocarcinoma cell lines. Cancers.

[121] Sun S., Jia Q., Zenova A.Y., Lin S., Hussainkhel A., Mezeyova J., Chang E., Goodchild S.J., Xie Z., Lindgren A. (2021). Identification of aryl sulfonamides as novel and potent inhibitors of Na _V_ 1.5. Bioorg. Med. Chem. Lett..

[122] Villalonga N., Ferreres J.C., Argilés J.M., Condom E., Felipe A. (2007). Potassium channels are a new target field in anticancer drug design. Recent Pat. Anti-Cancer Drug Discov..

[123] Arcangeli A., Crociani O., Lastraioli E., Masi A., Pillozzi S., Becchetti A. (2009). Targeting ion channels in cancer: A novel frontier in antineoplastic therapy. Curr. Med. Chem..

[124] Capatina A.L., Lagos D., Brackenbury W.J. (2022). Targeting ion channels for cancer treatment: Current progress and future challenges. Rev. Physiol. Biochem. Pharmacol..

[125] Djamgoz M.B., Onkal R. (2013). Persistent current blockers of voltage-gated sodium channels: A clinical opportunity for controlling metastatic disease. Recent Pat. Anti-Cancer Drug Discov..

[126] Brackenbury W.J., Isom L.L. (2008). Voltage-gated Na^+^ channels: Potential for beta subunits as therapeutic targets. Expert Opin. Ther. Targets.

[127] Djamgoz M.B.A., Mycielska M., Madeia Z., Fraser S.P., Korohoda W. (2001). Directional movement of rat prostate cancer cells in direct-current electric field: Involvement of voltage gated Na^+^ channel activity. J. Cell Sci..

[128] Yip D., Le M.N., Chan J.L.-K., Lee J.H., Mehnert J.A., Yudd A., Kempf J., Shih W.J., Chen S., Goydos J.S. (2009). A phase 0 trial of riluzole in patients with resectable stage III and IV melanoma. Clin. Cancer Res..

[129] Yang M., Brackenbury W.J. (2013). Membrane potential and cancer progression. Front. Physiol..

[130] Gould H.J., Miller P.R., Edenfield S., Sherman K.J., Brady C.K., Paul D. (2021). Emergency use of targeted osmotic lysis for the treatment of a patient with aggressive late-stage squamous cell carcinoma of the cervix. Curr. Oncol..

[131] Gould H.J., Edenfield S., Miller P.R., Sherman K.J., Melius B., Whitney A., Hunter R.P., Del Piero F., Tracey D., Paul D. (2022). The Role of Targeted Osmotic Lysis in the Treatment of Advanced Carcinoma in Companion Animals, A Case Series. Case Rep. Vet. Med..

